# Editorial: Perinatal Derivatives and the Road to Clinical Translation, Volume I

**DOI:** 10.3389/fbioe.2021.741156

**Published:** 2021-08-11

**Authors:** Antonietta R. Silini, Peter Ponsaerts, Ornella Parolini

**Affiliations:** ^1^ Centro di Ricerca E. Menni, Fondazione Poliambulanza- Istituto Ospedaliero, Brescia, Italy; ^2^ Laboratory of Experimental Hematology, Vaccine and Infectious Disease Institute (Vaxinfectio), University of Antwerp, Antwerp, Belgium; ^3^ Department of Life Science and Public Health, Università Cattolica del Sacro Cuore, Rome, Italy; ^4^ Fondazione Policlinico Universitario “Agostino Gemelli” IRCCS, Rome, Italy

**Keywords:** regenerative medicine, perinatal, secretome, consensus, *in vitro* characterisation

The International Network for Translating Research on Perinatal Derivatives into Therapeutic Approaches - SPRINT is a COST (Cooperation in Science and Technology) Action that brings together experts in terms of academic, clinical, and industrial knowledge from over 30 countries, in order to improve the basic understanding and the clinical translation of perinatal derivatives.

Perinatal tissues, and more specifically human placenta, has been traditionally used in Chinese medicine for centuries. Since the early 1900’s an increasing body of evidence has shown that these tissues have clinical benefits in a wide range of wound repair and surgical applications. The earliest reported applications of the placenta were on fetal membranes, and the first reports showing that the placenta also harbors cells which could have stem/progenitor properties, ultimately giving rise to their potential use in regenerative medicine, were published many years later (Bailo et al., 2004; Fukuchi et al., 2004; Igura et al., 2004; In ’t Anker et al., 2004; Soncini et al., 2007; Troyer and Weiss, 2008).

Nowadays, there is an undeniable need and desire to understand the mechanisms underlying the beneficial effects of perinatal tissues, and their derivatives such as cells and secretome, collectively referred to as perinatal derivatives (PnD). Many preclinical studies have now demonstrated that PnD may represent important tools for restoring tissue damage or promoting regeneration and repair of the tissue microenvironment. Despite a variety of PnD have been investigated in regenerative medicine approaches, their translation into clinical practice has been, to date, haphazard, incomplete and slow, ultimately limiting their therapeutic potential.

This Research Topic is dedicated to showcasing contributions that work toward a joint effort from the EU-funded COST SPRINT Action which addresses different issues that need to be faced in order to fully exploit the successful and efficient clinical applications of PnD, and to determine which PnD as well as it’s mode of application is optimal for defined diseases.

A major effort of the COST SPRINT Action broadly aims to approach consensus for different aspects of PnD research, such as providing inputs for future standards for the processing and *in vitro* characterization and clinical application of PnD. To this end, reference nomenclature for PnD must be established and consensus and universal guidelines for the donor eligibility, collection, culture, and cryopreservation should be defined. In this issue, Silini et al. propose consensus nomenclature for perinatal tissues and cells and address specific issues that are relevant for the definition/characterization of perinatal cells, starting from an understanding of the development of the human placenta, its structure, and the different cell populations that can be isolated from the different perinatal tissues. They also describe cell localization in the placenta and morphology and phenotype. Furthermore, Železnik Ramuta et al. provide several considerations for planning future studies and eventual translation of fetal membranes and their derivatives as antimicrobial agents from bench to bedside. These include the standardization of hACM, hAM and hCM preparation, standardization of the antimicrobial susceptibility testing methods, and designation of donor criteria that enable the optimal donor selection.

Strengthening a consensus approach, a detailed characterization of PnD is of utmost importance for the comparison of results in order to determine PnD efficacy in preclinical studies. Tehrani et al. describe biological features of the amniotic membrane and potential modifications in addition to the required processes for sterilization and preservation, such as combination with gels and other composites, and the preparation of amniotic membrane extract to tailor its use in regenerative medicine applications. In addition, Weidinger et al. break down the amniotic membrane into its different anatomical sub-regions and their properties such as morphology, structure, and content/release of certain bioactive factors. They discuss the relevance of these different properties for tissue regeneration, altogether helping in the optimization and fine-tuning of the clinical applications of the amniotic membrane.

The COST SPRINT Action also aims to review studies in animal models in order to grade efficacy of therapeutic interventions and identify research gaps for the disease of interest. Ultimately, understanding the therapeutic potential and underlying biological mechanisms will allow for the identification of which PnD could potentially provide the optimal results in specific diseases. In this issue, a particular focus has been made on the amniotic membrane. As a matter of fact, several groups discuss the application of the amniotic membrane in wound healing, one of it’s most advanced applications. Ruiz-Cañada et al. dissect the effects of the amniotic membrane on keratinocyte migration, proliferation and on the TGF-β signaling pathway and how this contributes to chronic wound healing. Vonbrunn et al. investigate the suitability of different scaffolds with amniotic membrane-derived mesenchymal stromal cells *in vitro* and *in vivo*, demonstrating potential new therapeutic approaches to wound care.

Janev et al. instead explore the multi-targeted anticancer activity of the homogenate of ammiotic membrane by reporting its effect on the morphology, adhesion, proliferation, cell cycle and ultrastructure of bladder cancer cells using 2D and 3D models. Their observations strongly encourage future studies to identify the molecules that induce the detrimental effects in cancer cells and their mechanism of action.

Odet et al. address the growing interest in human amniotic membrane in oral surgery, and they discuss in detail suitable procedures for it’s use in soft and hard tissue reconstruction in the oral cavity. This serves as a useful reference to guide new ideas for the development of innovative protective covering, suturing or handling devices in oral surgery. In addition, Etchebarne et al. present a systematic review of the literature to assess the benefit of using the amniotic membrane and derived products for bone regeneration. They underline how the amniotic membrane is a promising alternative to the commercially available membranes used for guided bone regeneration, and how cells isolated from the amniotic membrane can be combined with scaffolds for tissue engineering strategies applied to bone healing.

Other contributions focus on potential strategies to enhance perinatal cell therapeutic properties. For example, Zentelyte et al. investigated the effects of short term treatments of small molecules to improve the stem cell properties and differentiation capability of amniotic fluid stem cells. The results of this study provide valuable insights for the potential use of short term small molecule treatments to improve stem cell characteristics and boost differentiation potential of amniotic fluid stem cells. Citeroni et al. propose a new approach able to promote teno-differentiation for veterinary and medical purposes by evaluating the teno-inductive properties of the secretome derived from ovine tendon fetal tissue on ovine amniotic epithelial cells. They also discuss protocols for the production and storage of the optimal tendon-derived secretome.

Finally, two case studies present promising data for the use of lyophilized amniotic membrane in patients with chronic wounds. Schmeidova et al. performed a multicentre observational study on the use of a lyophilized amniotic membrane for the treatment of chronic wounds (various aetiologies). Out of 16 enrolled patients, 8 patients were completely healed, 6 patients demonstrated significantly reduced ulcer size and 2 subjects did not respond to therapy. This study demonstrates an effective alternative to the standard of chronic wounds care and confirms a significant effect of the application of lyophilized amntioic membrane for chronic wound management as a new standard of care. Lipový et al. present a case study using a lyophilized amniotic membrane for accelerating wound healing in a patient with Toxic epidermal necrolysis (TEN), a rare life-threatening disease that mainly affects the skin and mucous membranes, resulting from a toxic delayed-type hypersensitivity reaction type IV. Lyophilized amniotic membrane demonstrated excellent biocompatibility and accelerated epithelialization and the current therapy of patients with TEN with better outcomes and patient recovery.

Last, but not least, Papait et al. address a topic of recent and urgent interest, that is the COVID-19 pandemic. They provide and extensive overview of the characteristics of perinatal cells with a particular focus on the beneficial effects that they could have in patients with COVID-19, and more specifically for their potential use in the treatment of ARDS and sepsis.

This topic issue will be followed by a second volume that will further contribute to the SPRINT COST Action aimed to understand the mechanisms and therapeutic actions of perinatal derivatives, to critically discuss basic research data that can be useful for designing clinical trials, and to identify research gaps so as to guide future research on perinatal derivatives and streamline translation to the clinic.
